# Supplementary material for: RSV glycoprotein and genomic RNA dynamics reveal filament assembly prior to the plasma membrane
Source: Nat Commun. 2017 Sep 22;8:667. doi: 10.1038/s41467-017-00732-z (PMC5610308; doi:10.1038/s41467-017-00732-z)
Supplement: Supplementary file 1 — Supplementary Information [file 41467_2017_732_MOESM1_ESM.pdf]

File name: Supplementary Information
Description: Supplementary Figures and Supplementary Table

File name: Supplementary Movie 1
Description: RSV G on infected Vero cells was labeled with SBA-488 16 hpi and imaged live at 12.13 Hz. Movie is in real time.

File name: Supplementary Movie 2
Description: RSV G on infected Vero cells was labeled with SBA-488 12 hpi and was allowed to internalize for 1 h before imaging every 10 minutes. Images were recorded at 2 frames/s.

File name: Supplementary Movie 3
Description: RSV G on infected Vero cells was labeled with SBA-488 12 hpi and was allowed to internalize for 1 h before imaging live at 9 Hz to show filaments extension prior to nocodazole treatment. Movie is in real time.

File name: Supplementary Movie 4
Description: RSV G on infected Vero cells was labeled with SBA-488 12 hpi and was allowed to internalize for 1 h. After imaging briefly to confirm filaments extension, cells were treated with nocodazole and imaged live at 9 Hz. Movie is in real time.

File name: Supplementary Movie 5
Description: Cells were infected with RSV A2 for 12 hours before RSV genomic RNA was labeled with Cy3B MTRIPS at 30 nM (red). RSV G was then labeled with SBA-488 (green). After allowing 1 hour for internalization, cells were imaged live at 10 Hz. Movie is in real time.

File name: Peer Review File

Supplementary Information

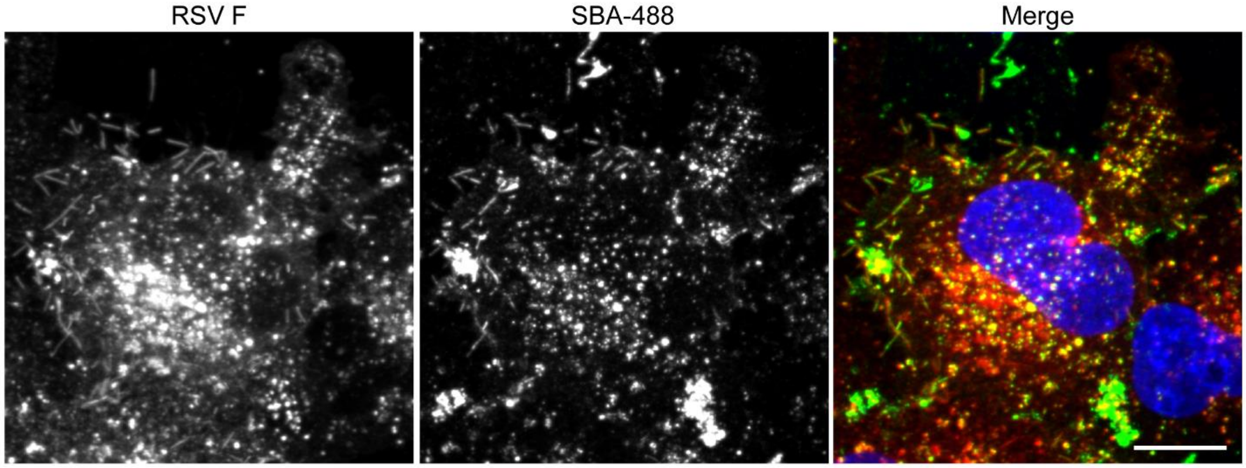

Supplementary Figure 1. Rapidly extending RSV G are RSV filaments. To confirm that the rapidly distending SBA-488 labeled structures were RSV filaments, the set of cells described in Fig. 6 for live-cell imaging was fixed and stained for RSV F (red) and nuclei (blue). Scale bar represents 10 μm.

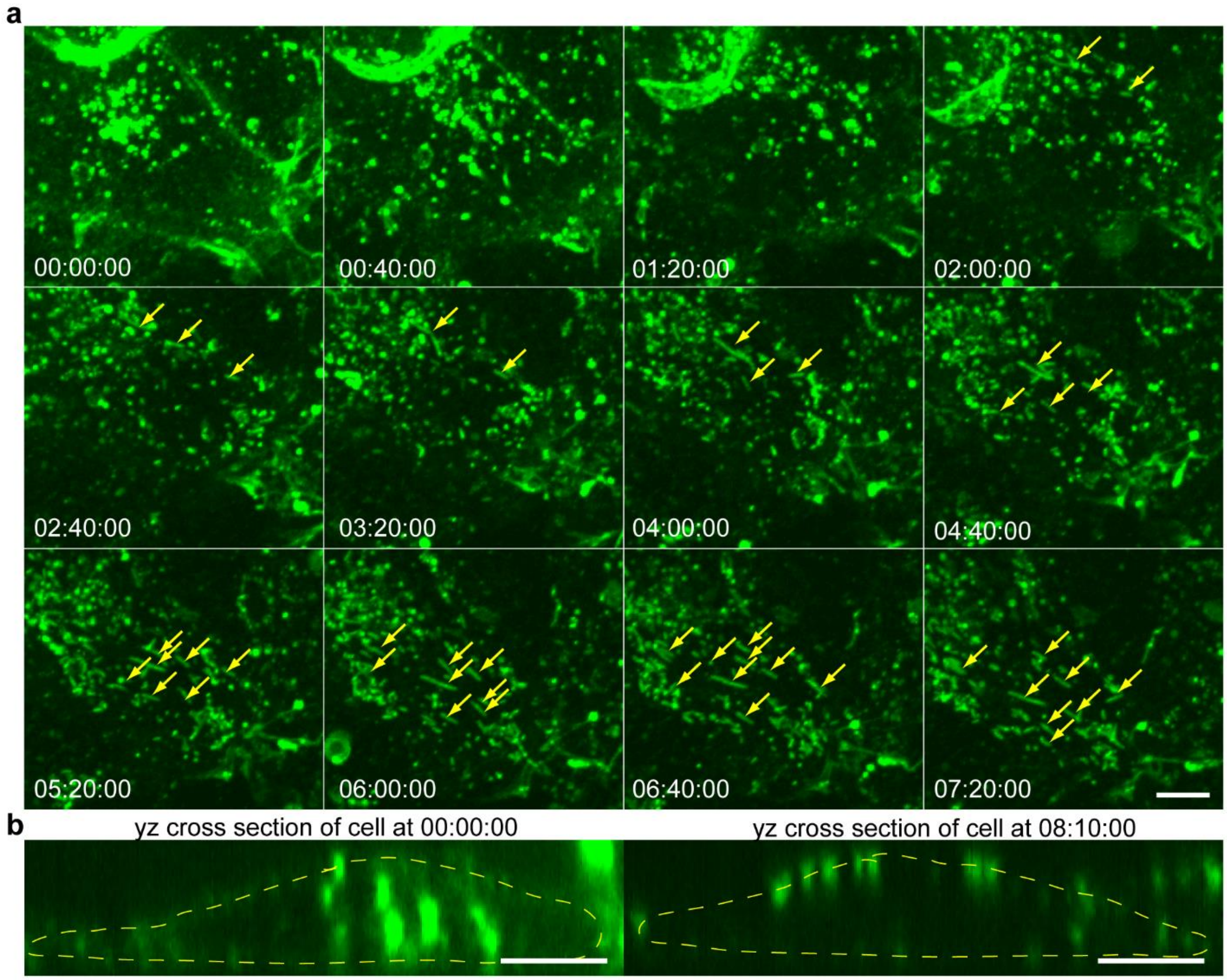

Supplementary Figure 2. RSV filaments are of formed by extension of RSV G vesicles. **(a)** Vero cells infected for 12 hpi were stained with SBA-488 (green) and imaged live every 10 minutes. Yellow arrows indicate progeny RSV filaments. Scale bar represents 10 µm. Cells are representative of duplicate experiments. **(b)** Cross-sectional views of the same cell 12 hpi and 20 hpi show RSV G granules moving from the intracellular compartment to the plasma membrane during the infection. The plasma membrane is represented by the yellow dashed line. Scale bar represents 10 µm.

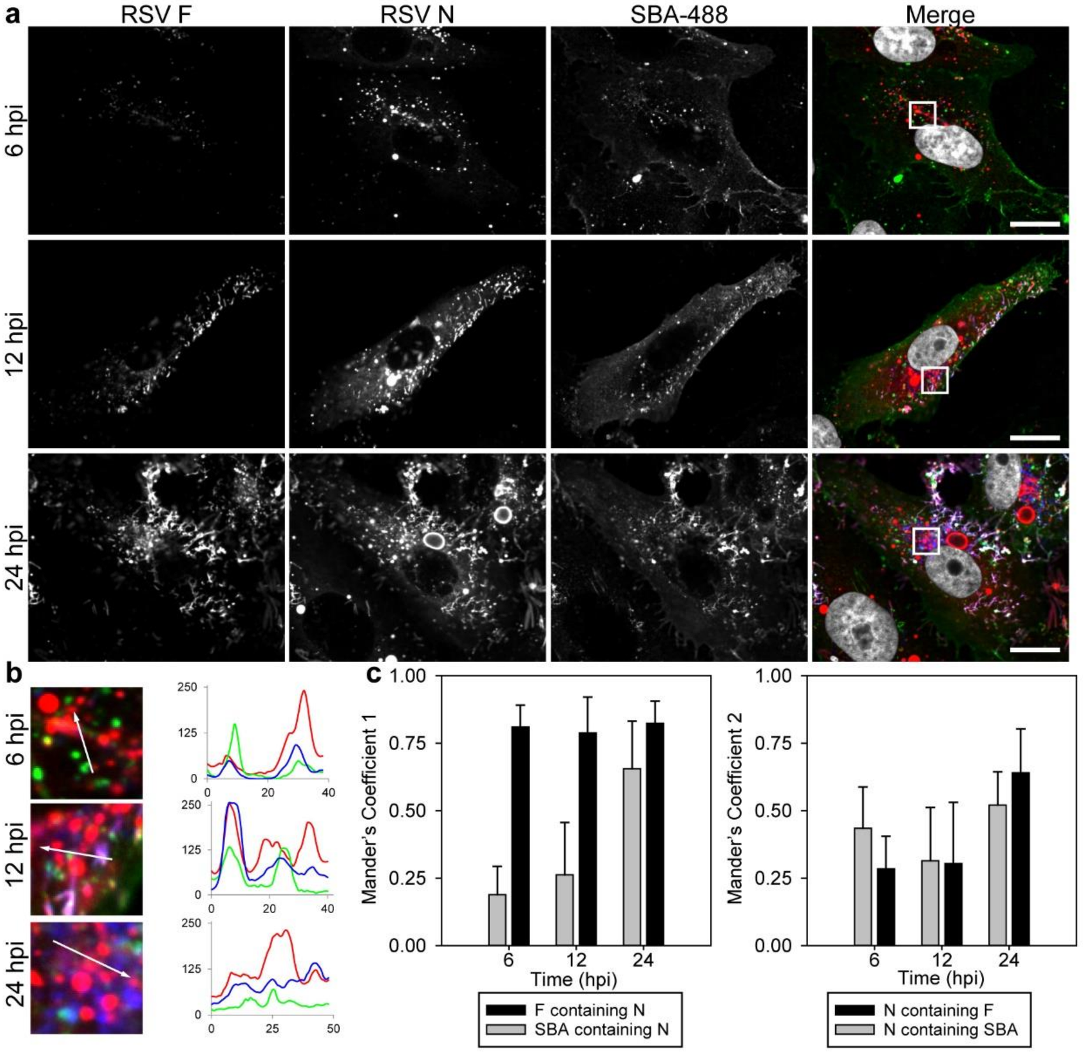

Supplementary Figure 3. RSV RNP granules merge with RSV G granules prior to the plasma membrane. **(a)** Vero cells were infected with RSV A2 at an MOI of 1. SBA-488 (green) was then delivered to the cells immediately prior to fixation at the indicated time-points post-infection. Cells were then stained for RSV F (blue), RSV N (red), and nuclei (gray). Scale bar represents 15 µm. **(b)** Enlarged cropped images from the white boxed regions from part **(a)** with intensity profiles showing colocalization along the direction of the white arrow. **(c)** Mander's colocalization analysis was performed on 30 individual cells per time-point. Data is represented as the mean Mander's coefficient per cell. Error bars represent standard deviation.

Gene	Forward Primer	Reverse Primer
RSV G	CGGCAAACCACAAAGTCACA	TTCTTGATCTGGCTTGTTGCA
RSV N	AAGGGATTTTTGCAGGATTGTTT	CTCCCCACCGTAACATCACTTG

Supplementary Table 1. qRT-PCR primers used in Figure 2.
